# Multi-year weed community dynamics and rice yields as influenced by tillage, crop establishment, and weed control: Implications for rice-maize rotations in the eastern Gangetic plains

**DOI:** 10.1016/j.cropro.2020.105334

**Published:** 2020-12

**Authors:** Khaled Hossain, Jagadish Timsina, David E. Johnson, Mahesh K. Gathala, Timothy J. Krupnik

**Affiliations:** a International Maize and Wheat Improvement Centre (CIMMYT), Sustainable Intensification Program, House 10/B. Road 53, Gulshan-2, Dhaka, 1213, Bangladesh; b Institute for Study and Development Worldwide, Sydney, 8/45 Henley Road, Homebush, West NSW, 2134, Australia; c Crop and Environmental Sciences Division, International Rice Research Institute (IRRI), Los Banos, Philippines

**Keywords:** Zero tillage, Direct seeding, Shannon-Weiner diversity index, Integrated weed management, Rank abundance

## Abstract

In South Asia's rice-based cropping systems, most farmers flood and repetitively till their fields before transplanting. This establishment method, commonly termed puddled transplanted rice (TPR), is costly. In addition, it is labor and energy intensive. To increase labor and energy efficiency in rice production, reduced or zero-tilled direct seeded rice (ZT-DSR) is commonly proposed as an alternative tillage and crop establishment (TCE) option. Effective management of weeds in ZT-DSR however remains a major challenge. We conducted a four-year experiment under a rice-maize rotation in Northwestern Bangladesh in the eastern Gangetic Plains to examine the performance of two TCE methods and three weed management regimes (WMR) on the diversity and competitiveness of weed communities in the rice phase of the rotation. The Shannon-Weiner Diversity Index, a measure of species diversity, was significantly greater under ZT-DSR than puddled TPR. It was also greater under no weed control (Weedy) and two manual weeding (MW) treatments compared to chemical herbicide with manual weeding (C + MW). In DSR Weedy plots, weed communities began shifting from grasses to sedges from the rotation's second year, while in the ZT-DSR and C + MW treatments, sedges were consistently predominant. In both puddled TPR Weedy and TPR C + MW treatments, broadleaves and grasses were dominant in the initial year, while sedges dominated in the final year. There were significant main effects of year (Y) and weed management regime (WMR), but not of TCE. Significant Y × TCE and TCE × WMR interaction effects on rice yield were also observed. Grain yields under ZT-DSR were similar to puddled TPR. ZT-DSR with one application of pre-emergence herbicide followed by one hand weeding at 28 days after establishment however resulted in significantly higher grain yield (5.34 t ha^−1^) compared the other weed management regimes. Future research should address methods to effectively manage weed community composition shifts in both ZT-DSR and TPR under rice-maize rotations utilizing integrated and low-cost strategies that can be readily applied by farmers in the eastern Gangetic Plains.

## Highlights

• A 4-year trial examined tillage and crop establishment (TCE) effects on weeds and rice yield.
• Zero-tilled direct seeded rice (ZT-DSR) yielded similarly as puddle transplanted rice (TPR).
• TCE had a large influence on weed community composition over time.
• Weed species diversity was greater with ZT-DSR than TPR.
• Safe, low-cost integrated weed management options are needed for ZT-DSR.

## 1 Introduction

The conventional approach to rice cultivation in much of South Asia is to flood and then repeatedly till fields before transplanting. These practices are commonly referred to as puddling, and lend a competitive advantage to rice seedlings over emerging weeds due to their relative size and developing canopy. Many weeds are also suppressed by repetitive tillage events. Standing floodwater can also inhibit germination (Rao et al., 2007). To reduce the risk of crop establishment problems and water losses from percolation, many South Asian farmers with lowland rice fields therefore prefer to grow flooded, puddled transplanted rice (TPR) (Chaudharya et al., 2017). In irrigated systems, repetitive wet tillage to puddle and prepare fields for transplanting can require large amounts of energy and water. Considering rainfed systems that have transitioned to mechanized cultivation over animal-traction based land preparation, puddled TPR can also require considerable tractor fuel and human energy (Sahrawat et al., 2010; Timsina and Connor, 2001). Puddled TPR also tends to require more time for crop establishment than direct seeded rice (DSR) (Gathala et al., 2014; Kumar and Ladha, 2011). For puddled TPR grown during the summer monsoon season, the additional time needed for preparing fields and transplanting causes delays in the crop cycle, setting back the rate at which farmers can harvest rice and prepare fields for subsequent winter (‘*rabi*’) winter season crops, including maize. Combined with repetitive tillage used to establish the maize crop, and delays in sowing after puddled TPR is harvested, maize yield losses of up to 22% can result relative to establishment within optimal dates. Such losses can occur due to heat stress and early monsoon season storm events that can cause waterlogging and lodging, and which become increasingly common as the winter shifts into the spring season (Ali et al., 2008). For these reasons, zero-tilled DSR (ZT-DSR) has been widely advocated as an alternative and ‘climate smart’ crop establishment method.

ZT-DSR has been reported to maintain yield, save water, reduce greenhouse gas emissions and production costs, thereby increasing farmers' potential to profit from rice cultivation (Ahmed et al., 2014; Alam et al., 2018; Chakraborty et al., 2017). Establishment of ZT-DSR during the monsoon season could also confer additional advantages to subsequently grown crops in the following season. This can be facilitated by enabling earlier ZT-DSR planting after the first rainfall event in the pre-monsoon, followed by earlier rice harvest, and rapid sequencing into rotated crops such as wheat or maize.

Modifications in crop establishment from puddled TPR to ZT-DSR can however result in considerable changes in weed community composition, density, and competitiveness with the crop (Chhokar et al., 2014; Singh et al., 2008). High weed pressure, low availability and high costs of appropriate herbicides, and inadequate integrated weed management strategies can reduce farmers’ adoption of ZT-DSR. In India, Singh et al. (2011) reported that rice yield losses due to uncontrolled weed growth were <12% with puddled TPR, compared to 85% and 98%, respectively, under conventionally tilled DSR (CT-DSR) and ZT-DSR. Weed biomass growth was also three times greater in DSR when established with CT or ZT compared to puddled TPR.

Many studies have reported that the dominance of particular weed species in rice cropping systems are significantly influenced by crop establishment method (Chhokar et al., 2014; Mahajan and Timsina, 2011; Mishra and Singh, 2012; Rao et al., 2007). Chauhan et al. (2012) reported that the density of grassy weeds was higher in ZT-DSR compared to puddled TPR. Sedges and broadleaves were conversely lower. Other studies (Kumar and Ladha, 2011; Rao et al., 2007) found annual grasses such as *Echinochloa colona* (L.) Link, *Digitaria ciliaris* (Retz.) Koeler and *Leptochloa chinensis* (L.) Nees*,* and perennial rhizomatous grasses such as *Cynodon dactylon* (L.) Pers., can have greater frequency and biomass under ZT-DSR. Conversely, broadleaves such as *Sagittaria guayanensis* Kunth, *Monochoria vaginalis* (Burm. f.) C. Presl ex Kunth, *Limnocharis flava* (*L.*) *Buchenau, Ludwigia octovalvis* (Jacq.) P.H. Raven*, Alternanthera sessilis* (L.) R. Br. ex DC. and *Ammannia baccifera* L. also had increased abundance in puddled TPR fields in Asia. Singh et al. (2005) also reported that the perennial sedge *Cyperus rotundus* L*.* and broadleaves such as *Commelina diffusa* Burm f. and *Caesulia axillaris* Roxb. are more common where tillage and crop establishment methods are changed from puddled TPR to CT-DSR.

Several studies suggest that changes in establishment methods from CT to ZT, or from TPR to DSR, resulted in changes in weed community composition and density (Chhokar et al., 2014; Kumar and Ladha, 2011; Kumar et al., 2008; Rao et al., 2007). Chauhan and Johnson (2009) observed that due to low soil disturbance in ZT-DSR, a large proportion of the weed seed bank on or near the soil surface remained undisturbed after sowing. Failure to manage these weeds can result in greater emergence of grasses and sedges including *D. ciliaris, E. colona* and *Eleusine indica* (L.) Gaertn., and broadleaves such as *Ageratum conyzoides* L., *Eclipta prostrata* (L.) L.*,* and *Portulaca oleracea* L. in ZT-DSR compared to CT-DSR. Kumar and Ladha (2011) reported that a shift from puddled TPR to ZT-DSR increased grass species richness and abundance, including *Dactyloctenium aegyptium* (L.) Willd., *L. chinensis, Eragrostis japonica* (Thunb.) Trin., and weedy rice, along with sedges such as *Cyperus rotundus* L., Fimbristylis quinquangularis (Vahl) Kunth, and *Cyperus iria* L. Similarly, Rao et al. (2007) also reported that after 12 years of direct seeding, weedy rice tended to be the dominant weed species, followed in order of importance by *Echinochloa crus-galli* (L.) P.Beauv., *L. chinensis*, and *Ischaemum rugosum* Salisb. Azmi and Baki (1995) also observed that changes in establishment method from puddled TPR to ZT-DSR shifted the dominance of weed species from broadleaves and sedges to grasses. This was attributed to the continuous use of herbicides in ZT-DSR.

Several pre- and post-emergence herbicides are now available for rice production in South Asia (Ahmed and Chauhan, 2014). A number of researchers have reported effective weed control can benefit yield in CT-DSR or ZT-DSR through the integrated use of pre- and post-emergence herbicides, rather than application individually (Mahajan and Chauhan, 2015; Mahajan and Timsina, 2011; Mishra and Singh, 2012; Rao et al., 2007). Mahajan and Timsina (2011) reported that the application of pre-emergence (pendimethalin) plus post-emergence (bispyribac) herbicides, followed by one manual weeding, can be a viable strategy to control weeds while increasing yield and profitability of CT-DSR in light-textured soils in North Western India. Mishra and Singh (2012) showed that pendimethalin followed by 2, 4-D significantly reduced *E. colona, C. iria* and *A. sessilis* densities, but did not control *C. axillaris*. Weed management strategies that combine the use of manual weeding and herbicides are likely to be more efficient than either manual weeding or herbicides alone (Ahmed et al., 2015). Integrated weed management approaches that employ preventive, cultural, mechanical and chemical methods are therefore necessary with ZT-DSR (Rao et al., 2007).

An improved understanding on the effect of weed community composition and dynamics on rice productivity could aid in the development of appropriate weed management options. This would include cropping systems that utilize direct seeding, and those in which the summer monsoon season rice crop is rotated with winter season alternative crops such as maize. Rice rotation with maize is an important double cropping system that is growing in popularity in the eastern Gangetic plains (EGP) of South Asia (Gathala et al., 2015; Krupnik et al., 2018; Timsina et al., 2011). Use of ZT-DSR instead of puddled TPR followed by maize established without tillage has been demonstrated as an option for cropping systems intensification. Application of conservation agricultural practices in rice-maize rotations has also been shown to reduce costs and increase profits without negatively affecting yield (Gathala et al., 2015). Considering these studies, potential shifts in weed communities and increased rice-weed competition that may arise from transitioning from puddled TPR to ZT-DSR. These issues therefore need to be studied to develop appropriate and integrated weed management strategies for rice grown in rice-maize rotations. In response to these challenges, the objectives of this study were to investigate weed community composition, population dynamics, and their implications for rice yield when comparing rice establishment and weed management methods within a multi-year rice-maize rotation in Northwest Bangladesh.

## 2 Materials and methods

### 2.1 Site characteristics

**Fig. 1:**
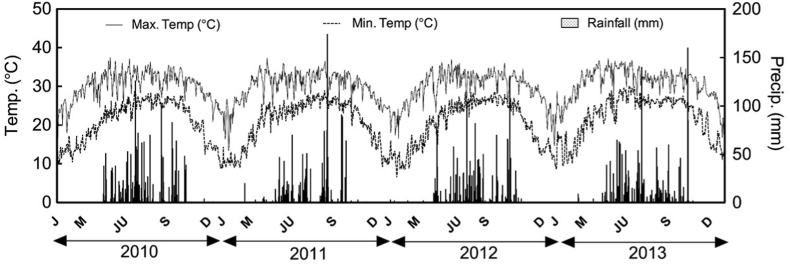
Monthly mean maximum and minimum temperatures (Temp.) and monthly precipitation (Precip.) during the study period (2010–2013) at the study location in Northwestern Bangladesh.

An experiment was conducted at the Bangladesh Rice Research Institute (BRRI) Research Station at Rangpur in North Western Bangladesh (25° 41ʹ45ʺ N; 89° 16ʹ 02ʺ E). Rangpur is under the Tista Meander Floodplain agroecological zone (Brammer, 2012). The soil (0–15 cm depth) was a sandy loam, with pH 6.44, organic carbon 0.86%, total N 0.3%, Olsen P 57 mol kg^−1^, exchangeable K 0.1 mol kg^−1^, exchangeable sulfur (S) 13 mg kg^−1^, and exchangeable zinc (Zn) 0.46 mg kg^−1^, measured following SRDI (2014). An automatic weather station located 4 km from the experiment recorded precipitation and temperature. Total rainfall across four years varied from 1265 mm in 2011 to 1475 mm in 2010; mean maximum and minimum temperatures respectively were 32.1 °C and 25.7 °C during the monsoon and winter season (Fig. 1).

### 2.2 Experimental design

The experiment was initiated in June 2010 with a monsoon season rice crop followed bywinter season maize. This rotation was maintained for three years followed by an additional rice crop in 2013, thus resulting in three maize and four rice crops. The experiment was laid out in a randomized split-plot design with four replications. Treatments were implemented only in the rice crop followed by a uniform maize crop grown using standard recommended practices in each year. The main and sub-plot sizes in rice were 133.92 m^2^ (21.6 m × 6.2 m) and 44.64 m^2^ (7.2 × 6.2 m), respectively.

Two tillage and crop establishment (TCE) methods were evaluated in main plots and three weed control methods in sub-plots. The main plot treatments were (1) zero-tilled direct seeded rice (ZT-DSR) and (2) puddled transplanted rice (TPR) while the sub-plot treatments were (1) weedy (no weed control), (2) manual weeding (MW) by hand hoe at 28 and 56 days after sowing (DAS) in ZT-DSR or transplanting (DAT) in puddled TPR, and (3) chemical and manual weed control (C + MW), achieved by applying a pre-emergence herbicide pendimethalin registered for use in DSR, followed by one manual weeding at 28 DAS or DAT, respectively. Glyphosate (isopropyl amine salt) at 1 L a.i. in 400 L water ha^−1^ was applied only to ZT-DSR C + MW treatment 3–5 days prior to crop establishment. Pendimethalin (formulation: emulsifiable concentrate or EC) was applied at 660 g a.i. ha^−1^ to a moist but unflooded soil in the ZT-DSR C + MW plots at 3 DAS, and in puddled TPR C + MW plots at 6 DAT. Sub-plot weed management treatments were not applied in the following maize crops. Rather, main plots with ZT-DSR and puddled TPR were rotated with zero-tilled and fully tilled maize, respectively. Maize following puddled TPR was weeded manually. Following glyphosate application one week prior to maize seeding, weeds were managed in zero tilled maize with one post sowing manual weeding. Weeds in the fully tilled maize were managed manually only.

### 2.3 Rice crop management

When there was sufficient soil moisture or precipitation to assure seed germination, ZT-DSR was established manually by dibbling seeds at a 20 cm × 15 cm hill-to-hill spacing using 30–35 kg seed ha^−1^. Establishment took place between 25 June and 7 July in each year. In puddled TPR, rice seedlings were raised by sowing seeds on seedbed between 15 May and 15 June, with two to three seedlings hill^−1^ transplanted between 15 June and 15 July (seedling age ranging from 25 to 30 days) in each year. Seedlings were transplanted to drained plots, after soil flooding and puddling, at 20 cm × 15 cm hill-to-hill spacing. A short-duration and popular cultivar, BRRI Dhan 33, was used. This variety matures between 105 and 110 days after sowing under direct seeding and between 115 and 120 days under transplanting (Rana et al., 2014). Irrigation was applied as needed to maintain at least a 5 cm floodwater layer in all plots equally, until at least two weeks before harvest. N, P, K, S and Zn fertilizers were applied at an elemental rate of 77, 6, 22, 7 and 3 kg ha^−1^, respectively, as urea, TSP, MoP, gypsum and zinc sulphate. All fertilizers except urea were applied as basal applications before tillage. In ZT-DSR, urea was broadcast in three equal splits (at 10, 25–30, and 45–50 DAS), while in puddled TPR, it was broadcast twice, at 15–20 and 35–40 DAT. There were no major incidences of insect pests or diseases, and hence no insecticides or fungicides were used. Weeds were managed in accordance with treatments as described above.

### 2.4 Data collection

This paper focusses only on the rice phase of the rice-maize rotation. A 0.5 m by 0.5 m quadrat was placed randomly in two locations in each sub-plot at 28 and 56 DAS or DAT in all treatments to measure weed density and biomass. All weeds inside the quadrat were carefully uprooted, cleaned, separated, and identified to species. Weed biomass was differentiated by species after removing root biomass with scissors and oven drying at 65 ^°^C for 72 h. Rice tillers were counted at 56 DAS or DAT, respectively, from a randomly placed 1 m^2^ surface in each sub-plot.

Rice grain and straw yields were determined from a sampling area of 10 m^2^ (5 m × 2 m) in the center of each sub-plot. Grain and straw were separated hand threshing. Rice yield was recorded at 14% moisture content. Straw yield was determined after oven drying a 1 m^2^ sub-sample drawn from the sub-plot straw harvest to 3% moisture content. Yield components were recorded from a randomly located 1 m^2^ surface per sub-plot located outside the area used for grain and straw sampling. Panicles were first counted from this area, after which ten panicles were randomly selected for measurements including panicle length, number of fertile and sterile grains, and 1000 grain weight.

### 2.5 Computations and analysis

The Shannon-Wiener diversity index (*H′*) characterizes species diversity in a community (Spellerberg, 2008). *H’* was calculated as described using the following equation:

(1) $$H^{'}=\;-\sum_{i=1}^{R}\text{ln}\left(pi\right)$$

where *R* is the number of species in the sample, Pi is the proportion abundance of a given species, and ln is the natural logarithm. Species diversity consists of two related components including richness (the number of species present in an observed area) and relative abundance (the density of one species relative to the density of the entire weed community) (Booth et al., 2003). We used rank abundance diagrams to represent species richness and species evenness. Richness is shown through the ranking of different species on the abscissa (Booth et al., 2003). Species evenness can conversely be inferred from the gradient of the line fitted in the abundance diagram. Steep gradients indicate unevenness, as high-ranking species have greater abundances than low-ranking species. A less steep gradient conversely indicates greater evenness within the community.

Four years of data from the rice crops in the rice-maize rotation were used for multi-year statistical analysis. Prior to analysis, all agronomic data were checked for normality and homoscedasticity. Data that failed to confirm to the assumptions of analysis of variance (ANOVA) were transformed using a square root transformation. Analysis proceeded using a three-factor ANOVA considering replication as a random factor using JMP (V11 software, Buckinghamshire, UK). Year of the experiment, crop establishment, weed control and their interactions were considered as fixed factors. The association of weed species with TCE treatments was explored by canonical correspondence analysis (CCA) using the vegan package (https://github.com/vegandevs/vegan) in ‘R-3. 5. 1’.

## 3 Results

### 3.1 Shifts in weed species and change in density

**Fig. 2:**
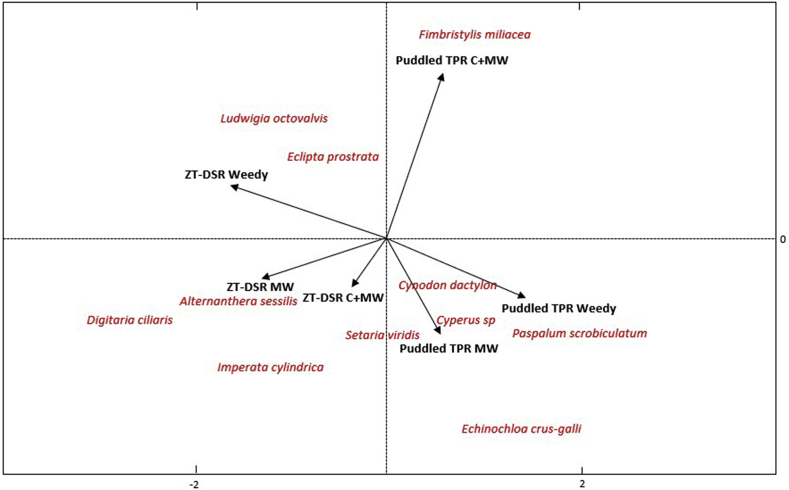
Canonical correspondence (bi-plot) analysis of weed communities (number of weed species) at 56 DAS/DAT in rice in a rice-maize rotation under different tillage and crop establishment and weed management options in the first year (2010) of experimentation in Northwestern Bangladesh. ZT-DSR = Zero-tilled direct seeded rice; TPR = Puddled transplanted rice; C + MW = Chemical followed by manual weeding; MW = Manual weeding. *Cyperus* (C.) sp. include *C. iria* and *C. difformis*.

**Fig. 3:**
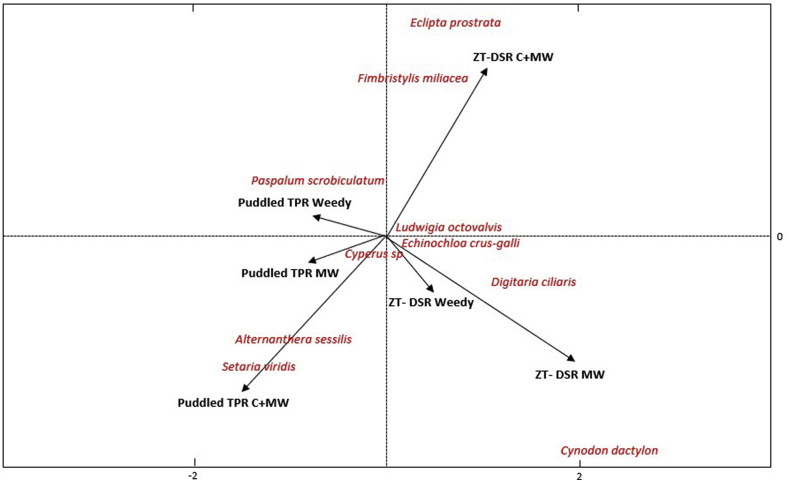
Canonical correspondence (bi-plot) analysis of weed communities (number of weed species) at 56 DAS/DAT in rice in a rice-maize rotation under different tillage and crop establishment and weed management options in the last year (2013) of experimentation in Northwestern Bangladesh. ZT-DSR = Zero-tilled direct seeded rice; TPR = Puddled transplanted rice; C + MW = Chemical followed by manual weeding; MW = Manual weeding. *Cyperus* (C.) sp. include *C. iria* and *C. difformis*.

Canonical biplots indicate that different weed species at 56 DAS or DAT, respectively in DSR and TPR, were associated with combinations of TCE and weed control methods during both the first (2010) and the last (2014) year of experimentation (Fig. 2, Fig. 3). In the first year, the density of *L. octovalvis* and *E. prostrata* (both broadleaves), were strongly associated with ZT-DSR weedy treatment, while *A. sessilis* and *D. ciliaris* (broadleaf and grass, respectively) had strong association with ZT-DSR with manual weeding (Fig. 2). After four years, weed species shifted to *E. crus-galli*, *C. difformis*, *C. iria*, and *D. ciliaris* in ZT-DSR weedy plots. A shift to *C. dactylon* in ZT-DSR MW plots, and to *E. prostrata* in ZT-DSR C + MW plots, was also observed (Fig. 3). In the puddled TPR weedy treatment, *Paspalum scrobiculatum* L. was prominent during both the first and last year of experimentation. Little change in community composition therefore occurred. In the puddled TPR MW treatment, initially three species (*C. dactylon*, *C. difformis* and *C. iria*) were most abundant, but after four years, *C. dactylon* could no longer be found, and only *C. iria* and *C. difformis* remained. In the puddled TPR C + MW treatment, weed species shifted from *F. miliacea* to *A. sessilis* and *Setaria viridis* (L.) P. Beauv. Furthermore, our observations indicated a strong association between puddled TPR MW and *E. crus-galli*. On the otherhand, though *S. viridis* (a grass) was associated with both ZT-DSR C + MW and puddled TPR MW in the first year, no such association was observed in the final year (Fig. 2, Fig. 3).

### 3.2 Shifts in weed species and change in biomass

There was a shift in weed composition over the four years of experimentation in both ZT-DSR and puddled TPR. Shifts were more pronounced in the former than the latter treatment. In the ZT-DSR weedy plots in the first year, grassy weeds including *D. ciliaris, C. dactylon*, and *F. miliacea* had greater biomass than other species. But from the second year onward, weed biomass was greater for *C. iria* and *C. difformis* and gramineous species such as *P. scrobiculatum and E. crus-galli*. In the ZT-DSR C + MW plots, weed biomass differed from that observed in ZT-DSR weedy plots, with *C. iria* and *C. difformis* dominating and producing greatest biomass, followed by *P. scrobiculatum* across all years. In the puddled TPR weedy plots, *P. scrobiculatum* was the most dominant species with greatest biomass across all years. Initially, *E. crus-galli* and *L. octovalvis* were the second and third in ranking in biomass, but from the second year forward, *C. iria, C. difformis and E. crus-galli* became more dominant species. In the puddled TPR C + MW plots also, *P. scrobiculatum* dominated in the first three years, but in the final year, *C. iria and C. difformis.* (sedges) were the most dominant weed species with greater biomass compared to the gramineous species *P. scrobiculatum* (Fig. 5).

### 3.3 Weed species diversity

Weed species diversity was significantly (*P* ≤ 0.001) affected by year, TCE and weed control options, although there were no significant interactions among these factors. The Shannon-Weiner Diversity Index showed that weed species diversity was significantly (*P* ≤ 0.001) higher in the final year of the experiment compared to earlier years (Table 1). Across the weed control methods, significantly greater (*P* ≤ 0.001) weed diversity was observed in ZT-DSR than puddled TPR. Weed control options also had a significant effect (*P* ≤ 0.001) on weed composition, with greater species diversity in the weedy and manually weeded treatments compared to the treatment that combined chemical and manual weeding (Table 1).

**Table 1:** ANOVA of the Shannon-Weiner Diversity Index for different tillage and weed management options in rice in a rice-maize rotation in Northwestern Bangladesh.

Variation sources		Shannon-Weiner Diversity Index
Year (Y)	2010	1.23 ab
	2011	0.99 c
	2012	1.06 bc
	2013	1.41 a
Tillage and crop establishment (TCE)	ZT-DSR	1.25 a
	Puddled TPR	1.09 b
Weed management regime (WMR)	Weedy	1.25 a
	MW	1.25 a
	C + MW	1.01 b
*F*-Values	Y	10.83***
	TCE	8.17***
	WMR	7.75***
	Y × TCE	1.94^NS^
	Y × WMR	0.86^NS^
	TCE × WMR	0.60^NS^
	Y × TCE × WMR	0.61^NS^

Different letters in a column indicate statistically significant differences according to Tukey's HSD at alpha = 0.05; ***, ** and * indicate significances respectively at *P* < 0.001, 0.01 and 0.05. NS indicates non-significance. ZT-DSR = Zero-tilled direct seeded rice; Puddled TPR = Puddled transplanted rice; Weedy = No weed control; MW = Two hand weeding; C + MW= Chemical followed by manual weeding.

### 3.4 Yield and yield components

Across treatments, rice grain yields were 0.45 t ha^−1^ greater (*P* ≤ 0.001) in the first and the fourth years compared to the second and third years (Table 2). Weed management regimes had a marked effect on rice yield. The C + MW treatment resulted in the highest (*P* ≤ 0.001) grain yield (5.10 t ha^−1^), followed by manually weeded (4.86 t ha^−1^) and weedy treatments (3.47 t ha^−1^). Considering interactions, TCE and weed management regime interacted (*P* ≤ 0.001), showing that yields for ZT-DSR with C + MW were greater than under puddled transplanted rice, with either C + MW or MW alone. Grain yield of ZT-DSR with two hand weedings was not significantly different from that of puddled TPR with C + MW. In the weedy plots, puddled TPR had significantly (*P* ≤ 0.001) greater yield (3.9 t ha^−1^) than ZT-DSR (3.0 t ha^−1^).

**Table 2:** Grain yield, yield components, and number of tillers at 56 days after seeding (DAS) or transplanting (DAT) as influenced as by tillage and crop establishment and weed control options in rice in a rice-maize rotation in Northwestern Bangladesh.

Variation sources	Grain yield (t ha^−1^)^a^	Panicles m^−2^	Panicle length (cm)	Sterility (%)	1,000 grain weight (g)	Tillers m^−2^^a^
Year (Y)						
2010	4.72 a	158.9	24.9 a	22.9 b	22.5 a	166.2
2011	4.22 b	156.5	24.8 ab	24.8 a	21.8 b	165.6
2012	4.33 b	154.1	24.2 b	24.2 ab	20.8 c	165.3
2013	4.64 a	151.0	24.2 b	23.7 ab	21.0 c	161.8

Tillage and crop establishment (TCE)						
ZT-DSR	4.48 a	152.8	24.8 a	23.4 b	22.1 a	162.9
Puddled TPR	4.48 b	157.5	24.2 b	24.4 a	20.9 b	166.5

Weed management regime (WMR)						
Weedy	3.47 c	132.7 b	24.1 b	24.7 a	20.4 b	143.4 b
MW	4.86 b	163.7 a	24.9 a	24.4 a	22.0 a	172.8 a
C + MW	5.10 a	169.0 a	24.5 ab	22.7 b	22.1 a	178.0 a

Y × TCE						
2010, ZT-DSR	4.63 ab	164.0 a	25.3	21.7	23.3	171.4 a
2011, ZT-DSR	4.36 abc	151.2 abc	25.1	24.6	22.4	161.0 b
2012, ZT-DSR	4.38 abc	148.7 bc	24.5	24.2	21.4	160.6 b
2013, ZT-DSR	4.53 ab	147.4 c	24.5	23.1	21.4	158.6 b
2010, Puddled TPR	4.81 a	153.9 abc	24.4	24.1	21.7	161.0 b
2011, Puddled TPR	4.08 c	161.9 ab	24.4	25.1	21.1	170.1 b
2012, Puddled TPR	4.27 bc	159.6 abc	23.9	24.3	20.2	170.0 b
2013, Puddled TPR	4.74 a	154.5 abc	23.9	24.3	20.6	165.0 b

TCE × WMR						
ZT-DSR Weedy	3.01 e	122.3 c	24.4	24.1 a	21.0 b	134.0 c
ZT-DSR MW	5.08 ab	164.0 a	25.2	24.6 a	22.6 a	173.9 a
ZT-DSR C + MW	5.34 a	172.0 a	24.9	21.4 b	22.8 a	180.8 a
Puddled TPR Weedy	3.93 d	143.0 b	23.8	25.2 a	19.8 c	152.7 b
TPR MW	4.63 c	163.4 a	24.6	24.2 a	21.5 b	171.7 a
Puddled TPR C + MW	4.86 bc	166.0 a	24.1	23.9 a	21.4 b	175.2 a

*F*-Values						
Y	10.6 ***	1.9 ^NS^	5.1 ***	3.9 ***	28.9 ***	0.7 ^NS^
TCE	0.0003 ^**NS**^	3.6 ^NS^	17.6 ***	6.2 ***	73.9 ***	2.3 ^NS^
WMR	190.7 ***	85.9 ***	8.6 ***	8.7 ***	58.8 ***	83.6 ***
Y × TCE	2.6 ***	4.1***	0.1 ^NS^	1.5 ^NS^	1.3 ^NS^	3.9 *
Y × WMR	1.0 ^NS^	1.7 ^NS^	0.2 ^NS^	1.0 ^NS^	0.8 ^NS^	1.7 ^NS^
TCE × WMR	39.2 ***	11.0 ***	0.1 ^NS^	3.8 ***	0.3 ^NS^	10.3 ***
Y × TCE × WMR	0.7 ^NS^	1.2 ^NS^	0.02 ^NS^	0.8 ^NS^	0.5 ^NS^	1.2 ^NS^

Different letters in a column indicate statistically significant differences according to Tukey's HSD at alpha = 0.05; ***, ** and * indicates significances at P < 0.001, 0.01 and 0.05, respectively; NS indicates non-significance.

ZT-DSR = Zero-tilled direct seeded rice; Puddled TPR = Puddled transplanted rice; C + MW= Chemical followed by manual weeding; MW = Two hand weeding; Weedy = No weed control; Tillers m^−2^ and panicles m^−2^ were counted respectively at 56 DAS/DAT and at harvest of rice.

a Data were square-transformed prior to analysis. Back transformed data are shown here.

Weed control options had a significant effect (*P* ≤ 0.001) on panicle density, with highest density in C + MW (169 panicles m^−2^) followed by MW (164 panicles m^−2^). ZT-DSR with C + MW had the greatest number of panicles (172.0 m^-2^), while ZT-DSR in weedy plots had the lowest (122 m^−2^). ZT-DSR had significantly (*P* ≤ 0.001) longer panicles than puddled TPR. The latter treatment conversely had significantly (*P* ≤ 0.001) greater 1,000 grain weight than ZT-DSR (22.1 vs. 20.9 gm). Weed control methods also had a significant effect (*P* ≤ 0.001) on tillers m^−2^ with C + MW producing the highest number of tillers (178 m^−2^) across treatments. ZT-DSR with C + MW also produced the greatest (*P* ≤ 0.001) number of tillers (181 m^−2^), whereas DSR weedy plots produced fewest (134 m^−2^).

## 4 Discussion

### 4.1 Effects of tillage and crop establishment and weed control methods on weed species and biomass and weed shifts

**Fig. 4:**
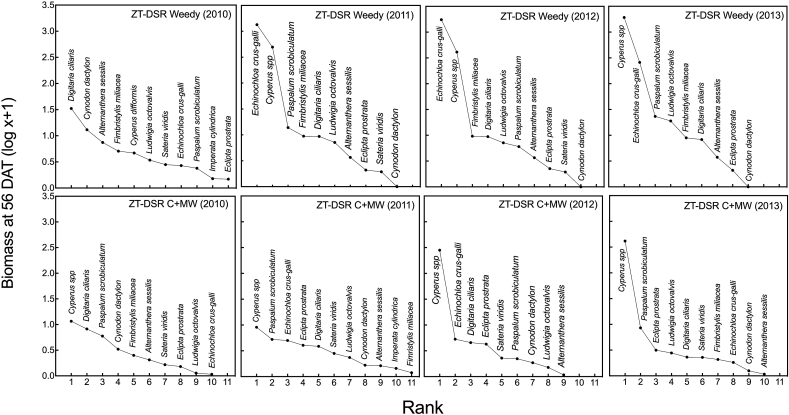
Abundance of weeds (dry biomass) at 56 DAS under three weed management options in zero-tilled direct seeded rice in rice-maize rotations from 2010 through 2013 in Northwestern t Bangladesh. C + MW = Chemical followed by manual weeding; Weedy = No weeding. *Cyperus* (C.) sp. include *C. iria* and *C. difformis*.

**Fig. 5:**
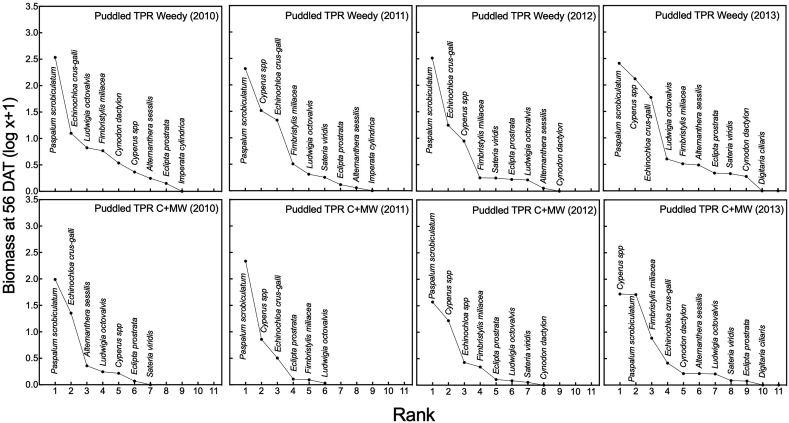
Abundance of weeds (dry biomass) at 56 DAT under three weed management options in puddled transplanted rice in rice-maize rotations from 2010 through 2013 in Northwestern Bangladesh. C + MW= Integrated weed management; Weedy = No weeding. *Cyperus* (C.) sp. include *C. iria* and *C. difformis*.

In this study, tillage and crop establishment and weed control methods exerted considerable influence on weed community composition and dynamics, in addition to the degree of weed competition with the crop (Fig. 2, Fig. 3, Fig. 4, Fig. 5). The pre-emergence herbicide pendimethalin, which was applied before seed sowing in each year, effectively controlled grasses but not sedges. Furthermore, continuous use of pendimethalin each year led to a population shift from grasses (e.g., *P. scrobiculatum, D. ciliaris* and *C. dactylon*) towards sedges (e.g., *C. difformis, C. iria and F. miliacea)* under the ZT-DSR C + MW treatment, although such shifts were more pronounced in ZT-DSR weedy plots. Yaduraju and Mishra (2004) found that continuous use of pendimethalin primarily controlled annual grasses and several broadleaved species, but not sedges. Weeds that had the greatest influence under ZT-DSR include *E. crus-galli, D. ciliaris*, *C. iria*, *C. difformis, L. octovalvis*, and *E. prostrata.* On the other hand, in the puddled TPR weedy plots, *P. scrobiculatum, C. difformis, C. Iria and E. crus-galli* were initially the most abundant weeds in terms of population and biomass. The dry weight of *E. crus-galli* was conversely lower compared to other weed species in the final year of rice cropping (Fig. 2, Fig. 3, Fig. 4, Fig. 5). This was due to intensive wet conventional tillage that was applied year after year in the puddled TPR plots. Seth et al. (1971) reported that intensive tillage and inadequate chemical weed control amplified *Paspalum distichum* L. density in rice in India. Our observations are also consistent with findings of Rao and Moody (1987) who reported that intensive tillage can reduce *Echinochloa glabrescens* Kossenko in rice.

Across years, we observed greater weed species and diversity and greater biomass and density under ZT-DSR than puddled TPR plots. In puddled TPR, wet tillage during land preparation and prior to rice transplanting adequately controls weeds, often without need for further weed management during the growing season (Kumar and Ladha, 2011; Rao et al., 2007). In contrast, in both CT-DSR and ZT-DSR, the lack of flood water during crop establishment favored weed growth over rice seedling growth. Furthermore, in the uncontrolled weedy plots, weed community diversity and biomass were greater in the ZT-DSR than the puddled TPR plots. These findings are consistent with several other studies in South Asia (Chauhan and Johnson, 2009; Mahajan and Timsina, 2011; Rabbani et al., 2011).

The findings of the current study supports previous studies indicating that weed community richness can be higher under CT-DSR and ZT-DSR than puddled TPR, even if weeds are controlled (Rao et al., 2007). In addition to a lack of significant mechanical disturbance during crop establishment, weed community richness in DSR is partially attributable to the absence of standing floodwater at the early stages of crop growth. Such conditions can facilitate the emergence and establishment of a diverse weed community (Chauhan et al., 2012). On the other hand, in puddled TPR, fields typically remain flooded or fully saturated, which suppresses the emergence of some weed species at early growth stages, and can be an important method of weed management (Kent and Johnson, 2001). Lastly, although not measured in the current study, higher rates of rice canopy development in the early stages of puddled TPR might have suppressed weeds, thereby lending a competitive yield advantage over DSR (Rao et al., 2007).

Several authors have reported that crop establishment using ZT-DSR can result in the development of dense weed seed banks close to the soil surface after sowing (Chauhan et al., 2012; Chauhan and Johnson, 2009; Mishra and Singh, 2012). Chauhan and Johnson (2009) found that when DSR was established using reduced tillage, 77% of the weed seed bank was found in the first 0–2 cm of soil, where they were more likely to be exposed to light. This contrasted with puddled TPR where weed seeds were buried at deeper depths. Ball (1992) found that when rice was established using puddling and transplanting, and when it was weeded by hand, that weed seeds and fragments of weed propagules spread laterally throughout the field through mechanical disturbance.

### 4.2 Effects of tillage and crop establishment and weed control methods on rice yield

This study showed no significant differences in rice yield between ZT-DSR and puddled TPR. The study however showed significantly higher grain yield with the C + MW treatment than with either two hand weedings or no weeding. The lack of significant difference in rice yield between ZT-DSR and puddled TPR can be attributed to good weed control achieved in the initial stage of both crops. This resulted in similar numbers of tillers and panicles m^−2^ in ZT-DSR and puddled TPR and significantly longer panicles, heavier grains, and reduced sterility in ZT-DSR compared to puddled TPR (Table 2). Norwood (1994) also reported that if crop stands are uniform and weeds are controlled successfully, then rice yields can be similar for ZT-DSR and puddled TPR. Achieving such precise weed control can however be challenging, and further consideration of the interactive effects of weed control on these treatments is crucial.

Weed competition in the early growth stage and a paucity of options for effective and affordable weed control in South Asia are two of the most important causes of yield losses in ZT-DSR (Gathala et al., 2014; Rao et al., 2017). In this study, weed biomass development was strongly associated with tillage and crop establishment. Where weeds were not controlled, rice yields were 32% lower under ZT-DSR compared to puddled TPR across weed management options and years. In ZT-DSR, where weeds were adequately controlled either manually or using the combination of chemical and manual methods, yields were slightly but not always significantly greater than their counterpart treatments in puddled TPR. Yields in weedy plots were however nearly 1 ton ha^−1^ lower under ZT-DSR than puddled TPR, providing evidence of the susceptibility of ZT-DSR to weed competition. These findings are consistent with Rabbani et al. (2011) who compared yields and weed species abundance in ZT-DSR and puddled TPR. Weed control during the early season of DSR is crucial to avoid large yield losses because young rice seedlings grow more slowly than rapidly establishing weeds that compete for light, water and nutrients (Chauhan and Johnson, 2009; Mahajan and Timsina, 2011). Rashid et al. (2012) reported that yield losses without weed control in wet seeded CT-DSR during its early growth stage range from 16 to 100%.

Across years, we observed that in puddled TPR, wet tillage during land preparation and prior to rice transplanting controlled weeds. This resulted in reduced effects on rice yield. In contrast, in both CT-DSR and ZT-DSR, the lack of flood water favored weed growth over rice seedling growth. Our results suggest that if weeds are not controlled in the early stage of DSR, yield losses are inevitable (Kumar and Ladha, 2011; Rao et al., 2007).

Farmers in Northwestern Bangladesh and similar areas of the EGP are increasingly cultivating maize in rotation with shorter-duration monsoon season rice varieties (Ali et al., 2008; Gathala et al., 2015). Delayed and uneven rainfall, in addition to high tillage costs for soil puddling during rice establishment, have been hypothesized as factors that may encourage farmers to consider reduced or zero-tilled DSR (Gathala et al., 2014; Kumar and Ladha, 2011). The usefulness of ZT-DSR as an energy-saving crop establishment method is however compromised by weed competition; poor weed management, for example, remains a key factor influencing farmers’ decision to abandon ZT-DSR after initial adoption (Chauhan, 2012; Mahajan et al., 2013). The results of our study indicate that ZT-DSR, along with one application of pre-emergence herbicide such as pendimethalin plus one hand weeding at 28 DAS, can effectively control grassy weeds and produce yields similar to puddled TPR. Rao et al. (2017) also reported that if weeds in ZT-DSR were controlled during the critical stage from seedling to panicle initiation, yields increased substantially. Monsoon season rice in Bangladesh and much of the EGP tends to be rainfed; under these conditions, sedges often predominate because of their high fecundity and rapid growth (Singh et al., 2008). Though pre-emergence herbicides are increasingly available to farmers, access to economic and environmentally sound options for control of sedges remains limited, indicating an important gap in research. The current study demonstrates that ZT-DSR with C + MW can be an alternative to puddled TPR in Bangladesh, with implications for similar environments across the eastern Indo-Gangetic Plains of South Asia. Further research is needed to identify more viable, cost-effective and environmentally sound C + MW options (i.e., using a range of low-toxicity and inexpensive pre- and post-emergence herbicides, mechanical or cultural methods, use of competitive rice cultivars, and methods to prevent weed seedbank development) that can be recommended to farmers seeking to reduce energy, time, and crop establishment costs while using ZT-DSR.

## 5 Conclusions and implications

This study examined effects of tillage and crop establishment coupled with weed control methods on weed richness and abundance in northwestern Bangladesh. To our knowledge, it is one of the first multi-year studies to document these effects in the context of a rice-maize rotation, which is a cropping system of increasing importance and popularity among farmers in South Asia. Our observations were that weed species diversity (as measured by the Shannon-Weiner Diversity Index) was greater in the fourth (final) year of experimentation compared to previous years, indicating increasing diversity over time. Weed diversity was also significantly greater in ZT-DSR compared to puddled TPR, and greater in the uncontrolled weedy treatment compared to one application of pre-emergent herbicide (pendimethalin) followed by one hand weeding. Across treatments, there was generally a shift from broadleaves and grasses (in the first year) to sedges (in the final year), although the richness and abundance of weed species varied across treatments.

Without the adequate control, grasses and sedges tended to dominate in ZT-DSR and broadleaves in puddled TPR. When herbicide and manual weeding is applied, sedges conversely dominate over broadleaves and grasses in ZT-DSR, underscoring the need for specialized weed control methods in these systems. Our results also reveal that effective control of grasses in DSR can be achieved by using pendimethalin along with one manual weeding at 28 DAS, although control of sedges remains a challenge requiring more research.

Grain yields of ZT-DSR with pendimethalin and one hand weeding was highest among tillage and crop establishment and weed control options. ZT-DSR yield with two hand weedings was however similar to puddled TPR with an application of pendimethalin and hand weeding. Hence despite the potential challenges posed by weed community composition shifts under directly seeded rice, our findings suggest that rice yields may be similar under ZT-DSR where effective control measures are applied in the ZT-DSR phase of a rice-maize rotation. Lastly, although this study demonstrates that there are viable options for weed control despite community composition shifts under multi-year rice-maize rotations with the use of direct seeded rice, further research is needed to document these observations in an on-farm research setting. In particular, rigorous studies of the economic impacts of these treatments on partial budgets and potential profits are needed, alongside ecotoxicological studies that consider the potentially negative implications of improper herbicide handling and application in the eastern Indo-Gangetic Plains.

## Credit author statement

KH contributed to methodology, formal analysis, investigation, data curation, writing of the original draft, and visualization. JT contributed to conceptualization, methodology, formal analysis, and writing the original draft, review and revision. DEJ contributed to conceptualization, methodology, analysis, writing review and revision, validation and funding acquisition. MKG contributed to conceptualization, methodology, formal analysis, writing review and revision and supervision. TJK contributed to methodology, validation, formal analysis, writing of the draft, reviews and editing, supervision, project administration and funding acquisition.

## Declaration of competing interest

The authors declare that they have no known competing financial interests or personal relationships that could have appeared to influence the work reported in this paper.
